# Elevated Prevalence of Abnormal Glucose Metabolism and Other Endocrine Disorders in Patients with β-Thalassemia Major: A Meta-Analysis

**DOI:** 10.1155/2019/6573497

**Published:** 2019-04-18

**Authors:** Li-Na He, Wei Chen, Yi Yang, Ying-Jun Xie, Ze-Yu Xiong, Di-Yu Chen, Dian Lu, Neng-Qing Liu, Ying-Hong Yang, Xiao-Fang Sun

**Affiliations:** ^1^Key Laboratory for Major Obstetric Diseases of Guangdong Province, Key Laboratory of Reproduction and Genetics of Guangdong Higher Education Institutes, The Third Affiliated Hospital of Guangzhou Medical University, Guangzhou 510150, China; ^2^Department of Urology, Zigong Fourth People's Hospital, Sichuan 643000, China

## Abstract

**Background:**

Endocrinopathies are common in patients with *β*-thalassemia major despite parenteral iron chelation therapy with deferoxamine. Prevalence of abnormal glucose metabolism in previous studies was controversial. The aim of this study was to discuss the prevalence of abnormal glucose metabolism in *β*-thalassemia major based on a meta-analysis.

**Methods:**

PubMed, ScienceDirect, Springerlink, Ovid, Web of Science, MEDLINE, Wanfang database, and Chinese National Knowledge Internet were searched for relevant articles. Two authors selected the articles according to the inclusion criteria and then extracted the data. The prevalence of diabetes mellitus (DM) in *β*-thalassemia major was defined as the primary outcome. The prevalence with the 95% confidence interval (95%CI) was used to evaluate the proportion of abnormal glucose metabolism and other endocrine disorders in patients with *β*-thalassemia major. Subgroup analyses were applied to explore the prevalence in different regions. Sensitivity analysis and publication bias assessment were also conducted.

**Results:**

A total of 44 studies with 16605 cases were included in this analysis. Diabetes mellitus was present in 6.54% (95% CI: 5.30%-7.78%). The fixed subgroup study revealed that the region with the highest prevalence was the Middle East (prevalence= 7.90%, 95% CI: 5.75%-10.05%). The accumulated meta-analysis revealed that the prevalence of DM in *β*-thalassemia major was relatively steady in each year. The prevalence of impaired fasting glucose (IFG), impaired glucose tolerance (IGT), and other endocrine disorders in *β*-thalassemia major was 17.21% (95% CI: 8.43%-26.00%), 12.46% (95% CI: 5.98%-18.94%), and 43.92% (95% CI: 37.94%-49.89%), respectively. Sensitivity analysis showed that the pooled results were robust; publication bias assessment revealed that there was no significant evidence that the pooled results were influenced by publication bias.

**Conclusion:**

High prevalence of endocrine disorders involving abnormal glucose metabolism was detected in *β*-thalassemia major. Treatment and prevention measurements may be necessary to prevent growth and endocrine problems.

## 1. Introduction

*β*-thalassemias are heterogeneous autosomal recessive hereditary anemia characterized by reduced or absent *β*-globin chain synthesis [1]. Iron overloading is frequently observed in *β*-thalassemia major patients with transfusion therapy [2]. Excessive iron can cause multiple organ damage when deposited in the liver, spleen, pancreas, heart, kidney, skin, pituitary, and other organs [3]. The complications related to iron overloading in patients with transfusion include myocardiopathy, congestive heart failure, liver cirrhosis, arthritis, endocrine disorders such as diabetes, and other diseases. In recent years, the prevalence of abnormal glucose metabolism in *β*-thalassemia major has gradually increased, which may be linked to increased life expectancy in patients with *β*-thalassemia major and growing concern about the combination of thalassemia with diabetes [4, 5]. However, according to previously published studies, the prevalence of abnormal glucose metabolism in *β*-thalassemia major was controversial. Hence, this study was conducted to perform a meta-analysis to assess the pooled prevalence of abnormal glucose metabolism in *β*-thalassemia major based on published studies.

## 2. Methods

### 2.1. Search Strategy

We aimed to search all published studies reporting abnormal glucose metabolism in *β*-thalassemia major patients. Due to the language limitations of the authors, this study only includes English and Chinese publications. To minimize bias, the study region was not being restricted. The search databases included PubMed, ScienceDirect, Springerlink, Ovid, Web of Science, MEDLINE, Wanfang database, and Chinese National Knowledge Internet (CNKI). The publication date was restricted to before June 2018. The present analysis complied with Meta-Analysis of Observational Studies in Epidemiology (MOOSE) guidelines. The search strategies in our analysis were as follows:

(#1) diabetes OR impaired glucose tolerance OR impaired fasting glucose
(#2) (*β* AND thalassemia) OR thalassemia OR Mediterranean anemia
(#3) (#1) and (#2)

To ensure a comprehensive analysis and minimize selection bias, all references of the included studies were manually retrieved and analyzed. All research and analysis procedures were conducted by two independent authors. Ambiguity in the analysis was verified by referring to the original paper.

### 2.2. Inclusion and Exclusion Criteria

The main inclusion criteria for our study were as follows. (i) Patients with *β*-thalassemia major were included if they fulfilled the clinical diagnostic criteria. (ii) All the patients were on regular blood transfusion. (iii) Only original research mentioning abnormal glucose metabolism which included diabetes mellitus (DM), impaired fasting glucose (IFG), or impaired glucose tolerance (IGT) was included. (iv) The diagnostic criteria of diabetes are based on WHO classification. However, several studies did not straightforwardly mention the word “WHO,” while the contents of diagnosis criteria in paper were in accordance with WHO. In this case, these papers also have been included in our analysis. (v) The results of the research were presented as calculated data that could be used to calculate the prevalence of different types of abnormal glucose metabolism. The exclusion criteria were as follows: (i) abnormal glucose metabolism was not well classified, in which data of DM, IGT, IFG, or other endocrine disorders cannot be independently extracted from original study; (ii) the data were reported in non-transfusion-dependent thalassemia patients; (iii) there were duplicate data or duplicate publications; (iv) the data could not be used to calculate the prevalence of abnormal glucose metabolism; (v) there was any type of commentaries, including systematic reviews, invited reviews, letters, response to editors, and conference abstracts; (vi) there were papers that were not peer-reviewed.

### 2.3. Data Extraction and Quality Assessment

Two authors independently selected trials and extracted information on design, selection criteria, numbers or prevalence of abnormal glucose metabolism in *β*-thalassemia major, and baseline assessments and quality assessment from each included study. Disagreements were resolved by discussion with other team members or contact with the original investigators who were all sent emails with requests for the exact data. For missing data that we could not obtain from the primary investigators, estimated values based on the Cochrane Handbook were used in our analysis. The methodological quality of each included study was assessed under the standard of previously published recommendations for observational studies. The checklist for assessing key terms was as follows: data collection method, potential bias of result, discussion of possible confounders, objectives of the research, baseline of patients, and statistical data. The total score was five. Any study with a score less than or equal to four was excluded as low quality.

### 2.4. Statistical Analysis

The meta-analysis was conducted using STATA software version 12.0 (STATA, College Station, TX, USA). We estimated prevalence with 95% confidence intervals for each study via OpenEpi software online, which is available at http://www.openepi.com/OE2.3/Menu/OpenEpiMenu.htm. Results from the “Wald (Normal Approximation)” method was used if np> 5 or n(1-p)> 5, where n represents the total number and p is the prevalence in the group. Otherwise (np≤5 or n(1-p)≤5), the Mid-P of the exact estimation method was selected. Heterogeneity was assessed using the chi-square and I^2^ statistics and considered significant at p values <0.05 for chi-square and >50% for I^2^. If I^2^ >50%, we conducted a sensitivity analysis to determine the reasons for heterogeneity. Due to the heterogeneity of unit of measurement, the difference of serum ferritin level between abnormal glucose metabolism group and normal glucose metabolism group was calculated as standard mean difference (SMD). When a significant Q-test with P<0.05 or I^2^>50% indicated evidence of heterogeneity, the random-effects model was performed. Otherwise, the fixed-effects model was implemented. In cases of heterogeneity, we applied the random-effects model instead of the fixed-effects model because the former includes both within-study variance and between-study sampling error in the assessment of the uncertainty of the results of a meta-analysis. The 95% confidence interval (CI) of each prevalence was calculated to identify the study outcome variation. An outcome measure is considered statistically significant when the 95% CI excludes zero. Missing data were sought from study authors; for data that were not obtainable, values were estimated using methods based on the prevalence of cases of abnormal glucose metabolism in *β*-thalassemia major. Egger's line regression and Begg's funnel plot were used to evaluate potential publication bias among the included studies. In addition, sensitive analyses and metaregression analysis were conducted to discuss the heterogeneity by publication year.

## 3. Results

**Figure 1:**
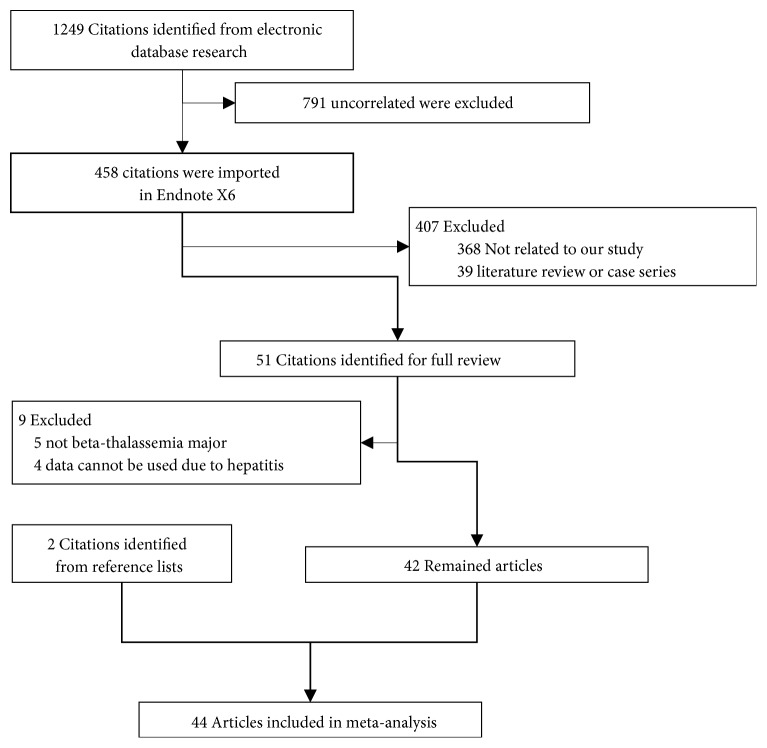
Study screening flow chart.

We searched for abnormal glucose metabolism and *β*-thalassemia major in PubMed, Ovid, CNKI, Wanfang Database, Embase, and Web of Science and obtained 1249 items. Of these studies, we determined that various studies were useless because they examined other diseases, deficiency with prevalence or interventions, and so on. Further screening selected 458 items suitable for reading of abstracts. Two studies [6, 7] were excluded due to the duplicated publication. Finally, of 51 full-text articles, only 42 studies were included in this meta-analysis [2–5, 8–45]. In addition, two studies were identified from reference lists [46, 47]. A total of 16605 cases diagnosed as *β*-thalassemia major were included in our study. The publication year ranged from 1994 to 2018. The selection flow chart is shown in Figure 1. Of the included studies, seven studies [30, 33, 34, 36, 39, 40, 43] were from Italy; seven studies [14, 16, 18, 20, 21, 31, 42] were from Iran; five studies [17, 19, 27, 44, 45] were from Egypt; five studies [4, 8, 22, 23, 35] were from the China region, including the mainland [22], Hong Kong [23], and Taiwan [4, 8, 35]; three studies [11, 12, 32] were from India; two studies [41, 47] were from Arabia; one study [37] was from Germany. The other studies were from Australia [26], Brazil [3], France [9], Lebanon [41], Oman [15], Turkey [25, 38], the United Kingdom [46], and the United States of America [2]. Eight studies [7, 14, 15, 19, 27, 29, 35, 39] reported the age at first diagnosis of abnormal glucose metabolism. 20 studies [2, 3, 9, 11, 12, 14, 15, 17, 19, 21–25, 27, 29, 35, 38, 41, 45] reported the serum ferritin level in *β*-thalassemia major patients. Baseline characteristics are listed in Table 1. As one of the most important indicators of endocrinopathy in *β*-thalassemia major, association between the serum ferritin levels and abnormal glucose metabolism was also determined based on 20 studies [2, 3, 9, 11, 12, 14, 15, 17, 19, 21–25, 27, 29, 35, 38, 41, 45]. When compared to normal glucose metabolism group, the mean serum ferritin level in abnormal glucose metabolism significant elevated (SMD=1.40, 95% CI: 0.44-2.36; Z=2.85, p=0.004).

### 3.1. Prevalence of DM in *β*-Thalassemia Major

**Figure 2:**
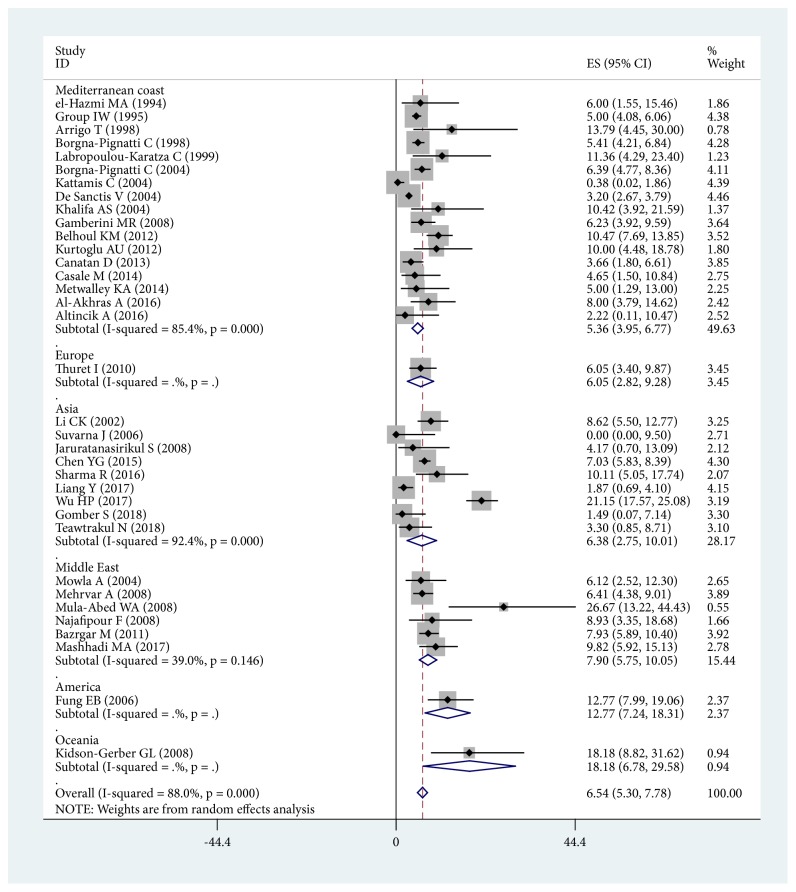
The overall prevalence and subgroup analysis of DM in *β*-thalassemia major.

A total of 35 studies were included which assessed diabetes mellitus. The publication year ranged from 1994 to 2018. The prevalence of original diabetes mellitus patients ranged from 0.00% to 26.67%. The meta-analysis revealed that the prevalence of diabetes mellitus in *β*-thalassemia major was 6.54% (95% CI: 5.30%-7.78%). A Forest plot is shown in Figure 2. In a subgroup analysis, the prevalence in the Mediterranean Coast was 5.36% (95% CI: 3.95%-6.77%). Nevertheless, the highest prevalence region was Oceania, with one study included (prevalence= 18.18%, 95% CI: 6.78%-29.58%) [26]. In order to avoid selection bias, regions with less than five studies were excluded [2, 9, 26]. The corrected subgroup study revealed that the highest-prevalence region was the Middle East (prevalence= 7.90%, 95% CI: 5.75%-10.05%). The subgroup analysis Forest plot is shown in Figure 2, with supporting data in Table 2.

### 3.2. Implication of Study Year for the Prevalence of DM in *β*-Thalassemia Major

**Figure 3:**
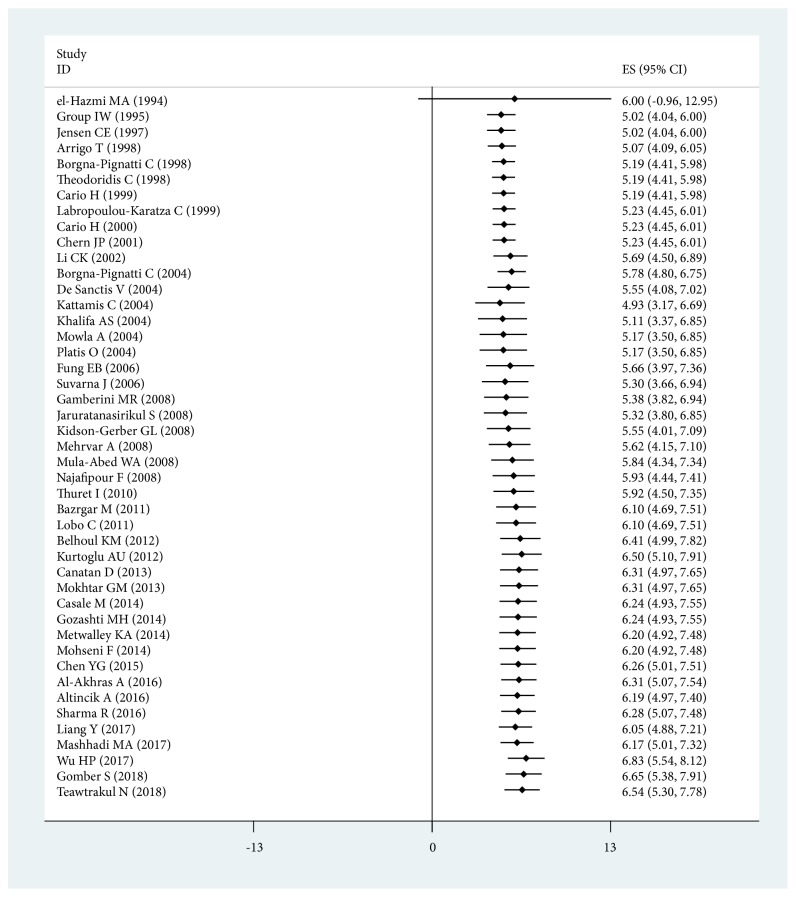
Accumulated meta-analysis for overall prevalence of DM in *β*-thalassemia major.

To assess the implication of study publication year for the prevalence of DM in *β*-thalassemia major, we analyzed the studies by time series. The lowest average prevalence was 2.39% (95% CI: 0.00%-4.93%) in 2018 reported by Gomber S [32] and Teawtrakul N [5], and the highest average prevalence was 11.36% (95% CI: 4.29%-23.40%) in 1999 reported by Labropoulou-Karatza C [24]. An accumulated meta-analysis for the prevalence of DM in *β*-thalassemia major was also conducted to assess the implication of publication year. The prevalence of DM was gradually increasing during the last 20 years, except in studies reported by Borgna-Pignatti C [39, 40], Kurtoglu AU [25], and Al-Akhras A [45] (Figure 3 and Table 3).

### 3.3. Prevalence of IFG in *β*-Thalassemia Major

**Figure 4:**
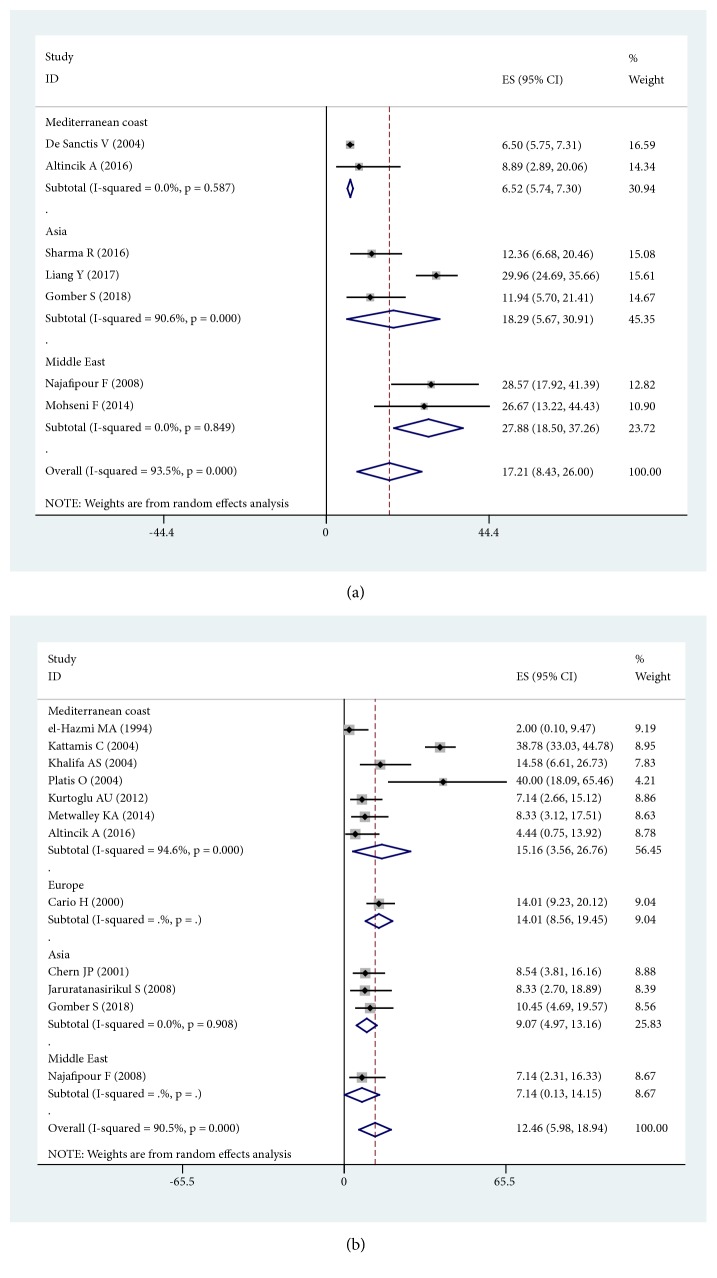
Prevalence of IFG and IGT in *β*-thalassemia major. (a) Prevalence of IFG in *β*-thalassemia major in different region; (b) prevalence of IGT in *β*-thalassemia major in different region.

Seven studies [12, 14, 18, 22, 32, 34, 44] examined impaired fasting glucose. The results of the analysis showed that the prevalence of IFG in *β*-thalassemia major was 17.21% (95% CI: 8.43%-26.00%). In a subgroup analysis, the prevalence of IFG in the Mediterranean Coast was 6.52% (95% CI: 5.74%-7.30%). The highest-prevalence region was the Middle East (prevalence= 27.88%, 95% CI: 18.50%-37.26%). The subgroup analysis Forest plot is shown in Figure 4(a), with supporting data in Table 2.

### 3.4. Prevalence of IGT in *β*-Thalassemia Major

Impaired glucose tolerance was inconsistently mentioned, and twelve studies were reported [13, 14, 19, 25, 27–29, 32, 35, 37, 44, 47]. The results of the analysis showed that the prevalence of IGT in *β*-thalassemia major was 12.46% (95% CI: 5.98%-18.94%). In a subgroup analysis, the region with the highest prevalence of IGT was the Mediterranean Coast (prevalence= 15.16%, 95% CI: 3.56%-26.76%). The subgroup analysis Forest plot is shown in Figure 4(b), with supporting data in Table 2.

### 3.5. Prevalence of Other Endocrine Disorders in *β*-Thalassemia Major

Eleven studies mentioned other endocrine disorders in *β*-thalassemia major. The main types were hypogonadotropic hypogonadism, hypothyroidism, and hypoparathyroidism. The results of the analysis showed that the prevalence of other endocrine disorders in *β*-thalassemia major was 43.92% (95% CI: 37.94%-49.89%). In a subgroup analysis, the region with the highest prevalence of other endocrine disorders was Europe (prevalence= 65.98%, 95% CI: 56.61%-75.35%), as shown in Table 2.

### 3.6. Sensitivity Analysis and Publication Bias Assessment

**Figure 5:**
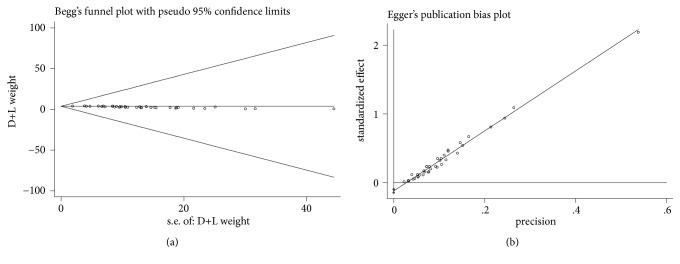
Publication bias assessment of included studies based on DM data. (a) Begg's funnel plot; (b) Egger's linear regression.

Sensitivity analysis was performed to evaluate the effects of the methodological quality of each trial on the pooled results. The number of cases in Arrigo T [43], Suvarna J [11], and Mula-Abed WA [15] was less than 30. The studies mentioned above were excluded. The sensitivity analysis showed that the pooled results were robust (prevalence=6.53%, 95% CI: 5.28%-7.79%). The publication bias for diabetes mellitus was detected with funnel plots (Figure 5(a)) and Egger's linear regression (Figure 5(b)). The shapes of the funnel plots and the linear regression showed no obvious asymmetry, indicating the absence of significant heterogeneity between these selected studies, and the pooled results were not influenced by publication bias.

## 4. Discussion

Thalassemia is common in the Middle East and Southeast Asia; it is an autosomal recessive hereditary disorder that is caused by various mutations or, rarely, deletions of *β*-globin gene on chromosome 11. The mutations lead to reduced or absent synthesis of the globin chain of hemoglobin, which causes imbalance between the alpha and non-alpha globin chains, therefore resulting in hemolysis for increasing of erythrocyte fragility [36]. Thalassemia has long-term extravascular hemolysis which increases iron absorption in the intestinal tract and decreases the bioavailability of iron. This phenomenon coupled with long-term multiple blood transfusions could lead to iron overload and increase the amount of iron ions [31, 32]. The most frequent endocrine disorders were abnormal glucose metabolism, hypogonadotropic hypogonadism, hypothyroidism, and hypoparathyroidism. Patients with thalassemia can exhibit pancreatic tissue and multiple organ dysfunctions. In a series of chronic malignant cycles, patients can develop type 1 diabetes caused by insufficient insulin secretion or type 2 diabetes caused by insulin resistance [48].

At present, bone marrow transplantation is the only available curative option for thalassemia major; due to the graft-versus-host disease and a lack of immunologically matched donors, the main treatment remains traditional long-term blood transfusion and Iron chelation therapy to maintain normal hemoglobin concentrations in the body. In addition, splenectomy is a treatment in thalassemia major with an increased blood requirement, hypersplenism, and symptomatic splenomegaly. These measures have dramatically improved the quality of life for patients with *β*-thalassemia major. But excessive iron deposition is also found in the pancreas, which leads to abnormal glucose metabolism [16, 32]. The pathogenesis of abnormal glucose metabolism may include the following aspects: (i) destruction of islet cells caused by excessive iron deposition, which results in insufficient insulin synthesis; (ii) excessive fatty acid oxidation induced by iron accumulation, with a decrease in the body's glycogen utilization rate; and (iii) reduction of hepatic capacity to uptake insulin caused by liver function damage [33, 34].

The results of the present study confirmed that the prevalence of diabetes mellitus, impaired fasting glucose, and impaired glucose tolerances in *β*-thalassemia major was 6.54%, 17.21%, and 12.46%, respectively. The results of the present study confirmed the fact that prevalence of abnormal glucose metabolism was gradually increasing during the last 20 years. There were no significant correlations between publication quality and the prevalence of DM in the publication bias or sensitivity analysis. Further analyses showed that the region with the highest prevalence of diabetes mellitus and impaired fasting glucose was the Middle East (7.90% and 27.88%, resp.). However, the region with the highest prevalence of IGT was the Mediterranean Coast (15.16%).

In previous reports, the prevalence of DM was reported to vary between 0.00% [11] and 26.67% [15]. This significantly wide interval may reflect differences in diagnostic criteria. The 95% confidence interval of diabetes mellitus in the present study is smaller than those in previous reports. This discrepancy may be due to the large number of cases included in our study compared to previous reports, which ranged from 15 to 3817 cases.

Moreover, we also found that 43.92% of *β*-thalassemia major patients were diagnosed with hypogonadotropic hypogonadism, hypothyroidism, and hypoparathyroidism. We could not evaluate the accuracy of the diagnosis of these endocrine disorders, which was a limitation of the original studies. Hence, we cannot comment on these diseases.

However, there were limitations in this analysis. First, significant heterogeneity was detected in the pooled analyses of prevalence. Although subgroup analyses with the addition of different regions were performed, the heterogeneity was not significantly reduced. This heterogeneity may be due to differences in the number of cases or basic characteristics. Second, there were insufficient data to conduct a complete evaluation of all regions around the world. Third, due to the deficiency of the original data, we could not perform further subgroup analyses by gender, age at diagnosis of endocrinopathy, serum ferritin, or length of blood transfusion. Studies have reported that either age at diagnosis of endocrinopathy or length of blood transfusion may significantly affect glucose metabolism as well as the serum ferritin level [28]. The presented evidence from published papers showed that glucose metabolism or endocrine disorder was also correlated with age or gender [12, 19]. These analyses could be considered in further studies based on newly reported data.

In conclusion, we identified the prevalence of abnormal glucose metabolism and other endocrine disorders in *β*-thalassemia major. Corresponding treatment and prevention measurements may be necessary to prevent growth and endocrine problems. These data should be dynamically updated as studies are published.

## Figures and Tables

**Table 1:** Baseline of included studies.

No.	Name	Year	Country	Number	Age	Age at diagnosis of AGM	Indicators	Quality	Serum ferritin
1	El-Hazmi MA	1994	Arabia	50	<15	NR	DM, IGT	5	NR
2	Group IW	1995	Italy	1861	1-40	NR	DM, EnD	5	NR
3	Jensen CE	1997	UK	97	NR	NR	EnD	5	NR
4	Arrigo T	1998	Italy	29	17-42	NR	DM	5	NR
5	Borgna-Pignatti C	1998	Italy	1146	NR	NR	DM	5	NR
6	Theodoridis C	1998	Greece	143	7-29	NR	EnD	4	NR
7	Labropoulou-Karatza C	1999	Greece	44	16-68(26.8±9)	NR	DM	5	2819±2450 ng/mL
8	Cario H	2000	Germany	157	1-37.5	NR	IGT	4	NR
9	Chern JP	2001	Taiwan, China	82	14.8±6.9	17.4(7-24)	IGT	4	3701.3±2530.7 ug/L
10	Li CK	2002	HK, China	232	1.4-30.3	NR	DM	5	5140(468-48490 pmoL/L)
11	Borgna-Pignatti C	2004	Italy	720	NR	7m(1d-3y)	DM	5	NR
12	Kattamis C	2004	Greece	263	11-14	NR	DM, IGT	5	NR
13	De Sanctis V	2004	Italy	3817	NR	NR	DM, EnD, IFG	5	NR
14	Khalifa AS	2004	Egypt	48	10-31(15.9±5.7)	16.8±4.5(11-23)	DM, IGT	5	3648±2219 ug/L
15	Mowla A	2004	Iran	98	8-32(15.9±4)	NR	DM	5	NR
16	Fung EB	2006	USA	141	25.8±8.1	NR	DM	5	3601±2351 ug/L
17	Suvarna J	2006	India	30	8-15	NR	DM	5	7623±2381 ng/mL
18	Mehrvar A	2008	Iran	437	34.7±1.4	NR	DM	4	NR
19	Jaruratanasirikul S	2008	Thailand	48	13.6±3.9	11-16	DM, IGT	4	5206±3291 ug/L
20	Kidson-Gerber GL	2008	Australia	44	over 18	NR	DM	4	NR
21	Gamberini MR	2008	Italy	273	13.9±1.4	NR	DM	5	NR
22	Mula-Abed WA	2008	Oman	30	16-32(21.23±3.42)	17.6±2.1(15-21)	DM, EnD	5	5647±3363 ug/L
23	Najafipour F	2008	Iran	56	15.62±4.44	19.8±4.3	DM, IGT, IFG	5	2888±948 ug/dL
24	Thuret I	2010	France	215	0.6-61.2	NR	DM	5	1240(89-7893) ng/mL
25	Farmaki K	2011	Greece	15	20-45(35±4.5)	NR	IGT, EnD	4	NR
26	Bazrgar M	2011	Iran	555	13.8±5.4	NR	DM	5	NR
27	Lobo C	2011	Brazil	960	28.5±19.8	NR	EnD	5	2000(405-15874) mcg/L
28	Kurtoglu AU	2012	Turkey	70	18.66±6.48	NR	DM, IGT	5	2350±450 ng/mL
29	Belhoul KM	2013	Arab	382	15.4±7.6	18±3.7	DM, EnD	5	2597.2±1976.8 ng/L
30	Mokhtar GM	2013	Egypt	447	0.9-35(14.2±7.7)	NR	EnD	5	1817±1399 ng/mL
31	Canatan D	2013	Turkey	246	15.3±8.6	NR	DM	5	4297.2±2122.5 ng/mL
32	Mohseni F	2014	Iran	30	5-19(14±4.4)	NR	IFG	5	NR
33	Casale M	2014	Italy	86	5-49(23.06±12.6)	NR	DM	5	NR
34	Gozashti MH	2014	Iran	300	NR	NR	EnD	5	NR
35	Metwalley KA	2014	Egypt	60	6-18	15.6±1.2	DM, IGT	5	3011±2101 mg/L
36	Chen YG	2015	Taiwan, China	1537	38.2±14.1	NR	DM	5	NR
37	Al-Akhras A	2016	Egypt	100	12-18(14.2±1.37)	NR	DM	5	3577.5±1826 mg/L
38	Sharma R	2016	India	89	13.6±2.5	NR	DM, EnD, IFG	5	6667±3294 pmoL/L
39	Altincik A	2016	Egypt	45	12-18(14.2±1.37)	NR	DM, IGT, IFG	5	NR
40	Mashhadi MA	2017	Iran	163	17.80±5.27	NR	DM	5	4563.50±2849.30 ng/mL
41	Liang Y	2017	China	267	7.9±0.2	NR	DM, IFG	5	4476±158 ug/L
42	Wu HP	2017	Taiwan, China	454	0.1-48	NR	DM	5	NR
43	Gomber S	2018	India	67	7.43±4.48	NR	DM, IGT, IFG	5	NR
44	Teawtrakul N	2018	Thailand	91	19.5±10	NR	DM	5	NR

NR: not reported; DM: diabetes mellitus; IFG: impaired fasting glucose; IGT: impaired glucose tolerance; AGM: abnormal glucose metabolism.

**Table 2:** Prevalence of abnormal glucose metabolism and other endocrine disorders in *β*-thalassemia major classified by study region.

Region	DM		IFG		IGT		EnD	
	P (%)	95% CI (%)	P (%)	95% CI (%)	P (%)	95% CI (%)	P (%)	95% CI (%)
Mediterranean coast	5.36	3.95-6.77	6.52	5.74-7.30	15.16	3.56-26.76	39.82	33.55-46.10
Middle East	7.90	5.75-10.05	27.88	18.50-37.26	7.14	0.13-14.15	36.67	31.22-42.11
Europe	6.05	2.82-9.28	NR	NR	14.01	8.56-19.45	65.98	56.61-75.35
America	12.77	7.24-18.31	NR	NR	NR	NR	27.50	24.67-30.32
Asia	6.38	2.75-10.01	18.29	5.67-30.91	9.07	4.97-13.16	55.06	44.80-65.32
Oceania	18.18	6.78-29.58	NR	NR	NR	NR	NR	NR
Overall	6.54	5.30-7.78	17.21	8.43-26.00	12.46	5.98-18.94	43.92	37.94-49.89

DM: diabetes mellitus; IFG: impaired fasting glucose; IGT: impaired glucose tolerance; EnD: endocrine disorders; P: prevalence; 95% CI: 95% confidence interval; NR: not reported.

**Table 3:** Prevalence of abnormal glucose metabolism and other endocrine disorders in *β*-thalassemia major (%).

Year	DM	IFG	IGT	EnD
1994	6.00(0.00-12.96)	NR	2.00(0.00-6.68)	NR
1995	5.00(4.01-5.99)	NR	NR	49.01(46.74-51.28)
1997	NR	NR	NR	65.98(56.61-75.35)
1998	7.09(0.51-13.67)	NR	NR	28.67(24.28-33.06)
1999	11.36(1.81-20.92)	NR	14.14(9.27-19.01)	NR
2000	NR	NR	14.01(8.57-19.46)	NR
2001	NR	NR	8.54(2.37-14.72)	NR
2002	8.62(4.99-12.26)	NR	NR	NR
2004	4.01(1.63-6.39)	6.50(5.72-7.28)	18.15(9.99-26.32)	40.57(38.73-42.40)
2006	6.30(0-18.82)	NR	NR	NR
2008	7.89(4.63-11.14)	28.57(16.84-40.31)	7.65(2.35-12.95)	73.33(57.73-88.94)
2010	6.05(2.82-9.28)	NR	NR	NR
2011	7.93(5.68-10.19)	NR	NR	27.50(24.68-30.33)
2012	10.39(7.57-13.23)	NR	7.14(0.91-13.37)	31.94(27.27-36.61)
2013	3.66(1.26-6.07)	NR	NR	44.74(40.14-49.35)
2014	4.79(1.14-8.44)	26.67(11.06-42.27)	8.33(1.14-15.53)	36.67(31.23-42.12)
2015	7.03(5.75-8.31)	NR	NR	NR
2016	6.54(1.87-11.22)	11.00(5.63-16.37)	4.44(0-11.03)	55.06(44.81-65.32)
2017	10.87(0-23.28)	29.96(24.47-35.45)	NR	NR
2018	2.30(0-4.93)	11.94(4.08-19.79)	10.45(3.01-17.89)	NR

DM: diabetes mellitus; IFG: impaired fasting glucose; IGT: impaired glucose tolerance; EnD: endocrine disorders; NR: not reported.
